# Management of Dental Patients With Mental Health Problems in Special Care Dentistry: A Practical Algorithm

**DOI:** 10.7759/cureus.34809

**Published:** 2023-02-09

**Authors:** P Poornachitra, Vivek Narayan

**Affiliations:** 1 Oral Medicine, Diagnosis, and Radiology, Saveetha Dental College and Hospital, Chennai, IND

**Keywords:** mental health service, public health dentistry, dental clinics, mouth diseases, oral health

## Abstract

Introduction

Individuals with mental health problems have poor oral health affecting their quality of life with an increased burden on their well-being. Dentists find treating them challenging due to a lack of training and awareness in modifications of dental care delivery in special needs patients. Also, polypharmacy is common in psychiatric care, further complicating dental care while prescribing routine medications as potential drug interactions must be considered.

Methods

Due to a lack of clinical practice guidelines in the literature and the absence of guidelines issued by dental governing bodies, we attempted to consolidate the existing challenges and propose a model for managing psychiatric special needs patients.

Results and discussion

Based on the current evidence, we hereby recommend ‘psychiatric dental consultation liaison’ (PDCL) services as the acceptable framework for the management of dental patients with mental health problems in special care dentistry.

Conclusion

PDCL services will favour both dentists and patients as it includes psychiatric consultation and interventions that will result in the positive execution of comprehensive dental treatment care.

## Introduction

'Special needs' or 'special care' patients refer to individuals whose physical, emotional, social, and intellectual skills fall remote of the accepted normal in regards to the existing standards of growth and development and who require special and/or supplementary care throughout their life [1]. Globally, as per the World Health Organization (WHO), one in every eight people lives with mental health problems. The Comprehensive Mental Health Action Plan of 2013-2030 issued by the WHO recommends that people with psychiatric problems should not be stigmatized and discriminated against in their access to high-quality healthcare services to promote their health recovery [2]. In general, mental health is described as a level of well-being in which the individual identifies the skills to cope with routine stressful situations of life and can work effectively and fruitfully by making adequate contributions to society. The indicators of good mental health include emotional, psychological, and social well-being with the ability to learn, manage emotions, build relationships, and cope with uncertainty. Mental or psychiatric illness is referred to as health conditions that are manifested by changes in perception, thinking, behaviour, or mood, or in a combination of all the factors that are related to distress and/or limited functioning [3].

There has been a recognized correlation between dental health and quality of life and self-respect. Oral diseases in patients with mental health problems are often overlooked due to patients’ lack of motivation, lack of awareness, poor economic status, phobias, and unwelcoming attitudes by dentists. Hence, this article aims to create a set of clinical practice guidelines for dentists in treating patients with mental health disorders as not much substantial information is currently available in the literature. The objective is to bridge the knowledge gap among dental practitioners and establish a multisectoral treatment approach in delivering oral health care.

## Materials and methods

The International Statistical Classification of Diseases and Related Health Problems, 10th revision (ICD-10) categorizes mental and behavioural disorders using certain codes for standardization in referencing (Table 1) [4]. Based on the available literature, we could summarize the general and behavioural aspects of individuals with mental health problems as in Table 2. Based on the available updates, we have proposed our algorithm for the management of dental problems in mental health disorder patients who report for dental care.

**Table 1:** International Statistical Classification of Diseases and Related Health Problems: F00–F99: Mental and Behavioral Disorders

Code	Description
F00–F09	Organic, including symptomatic, mental disorders
F10–F19	Mental and behavioural disorders due to psychoactive substance use
F20–F29	Schizophrenia, schizotypal and delusional disorders
F30–F39	Mood [affective] disorders
F40–F48	Neurotic, stress-related, and somatoform disorders
F50–F59	Behavioural syndromes associated with physiological disturbances and physical factors
F60–F69	Disorders of adult personality and behaviour
F70–F79	Mental retardation
F80–F89	Disorders of psychological development
F90–F98	Behavioural and emotional disorders with onset usually occurring in childhood and adolescence
F99	Unspecified mental disorder

**Table 2:** Dental Aspects of Mental Health Problems

Mental health problem	General and behavioural aspects to be considered in dental clinics
Anxiety and stress	
Anxiety disorders	Previous frightening dental experience; fear of noise and vibration of drill, site of injection, sitting on treatment chair; hostile response; white knuckle syndrome: tense, agitated, incessant chatter, failed dental appointments
Stress	Low levels of steroid; dehydroepiandrosterone (DHEA); impaired immunity, cardiovascular disease, Alzheimer's disease, hypothyroidism, and diabetes
Post-Traumatic Stress Disorder (PTSD)	Patients present with varied unexplained pain problems
Generalised Anxiety Disorder (GAD)	Physical symptoms, such as fatigue, headaches, muscle tension, muscle aches; difficulty in swallowing; trembling, twitching, and irritability; sweating and hot flushes; trouble falling or staying asleep
Panic disorders	Features of catecholamine release: the heart pounds and the patient may feel sweaty, weak, faint or dizzy; hyperventilation
Social Anxiety Disorder	Blushing, profuse sweating, trembling, nausea, and difficulty in talking
Phobic Neuroses	Claustrophobia (fear of closed spaces)
Personality Disorders	
Borderline Personality Disorder	Aggression; mood instability; impulsive behaviour; missing dental appointments or to pay much attention to oral health-care instructions; treatment plans may be argued about or frankly refused; payment may be withheld; litigation threatened
Obsessive Compulsive Disorder (OCD)	Obsessions centred on the mouth, compulsive tooth-brushing, excessive use of antiseptic mouthwashes; obsessed with the possibility of infections or cancer
Childhood Disorders	
Attention Deficit Hyperactivity Disorder (ADHD)	Uncontrolled activity; impulsiveness; impaired concentration; motor restlessness; extreme fidgeting
Somatization Disorders	
Somatization Disorder	Absence of organic cause or physical signs; persistence of symptoms for very long periods, sometimes for years; bizarre symptoms such as ‘powder’ or ‘slime’ coming out of the painful area; vague localization of symptoms; unrecognizable stimuli; multiple unexplained symptoms presented in colourful, exaggerated terms; rejects prescribed drugs; overtly depressed
Hypochondriasis (Health anxiety)	Complaints of dry or burning mouth, disturbed taste, and oral or facial pain; presentation of the medical history in excessive detail; absence of organic disease or physiological disturbance; unwarranted fears or ideas persisting despite reassurances; clinically significant distress; complaints not restricted to appearance or delusional intensity; negative physical examination and laboratory results
Conversion Disorder (Hysteria)	Symptoms preceded by stress Neurological, medical, substance abuse or cultural explanation Severe distress Absent or insufficient significant laboratory findings
Somatization Pain Disorder (Briquet syndrome)	Physical symptoms that mimic disease or injury, for which there is no identifiable physical cause
Body Dysmorphic Disorder (BDD)	Compulsive checking in mirrors, windows, and other reflective surfaces attempts to camouflage the imagined defect; excessive grooming; compulsive skin-touching; reassurance-seeking; compulsive information-seeking; obsession with cosmetic procedures, such as orthognathic or plastic surgery, with few satisfactory results for the patient
Malingering	Deliberate simulation or exaggeration of symptoms for obvious and understandable gain
Factitious Injuries	Bruxism; cheek-biting or the buccal mucosa ((morsicatio buccarum); exfoliative cheilitis; eelf-extraction of teeth; gingiva injury by picking with fingernails; caustic substances application to lips
Hysterical Neuroses	Physical complaints that have no demonstrable organic basis, such as pain, anaesthesia, dysphagia, fainting, fits, paralysis or tremor, but which frequently result in patients submitting to repeated operations
Munchausen Syndrome	Common complaints are acute abdominal pain, fever, haematuria, or infected wounds.
Compensation Neurosis	Usually follows accidents like head injury; rapid disappearance of symptoms with the settlement of the claim
Self-Harm	
Self-Harm (SH) or Deliberate Self-Harm (DSH)	Skin-cutting; burning; scratching; banging or hitting body parts Interfering with wound healing; hair-pulling (trichotillomania), ingestion of objects (pica), or toxic substances
Psychiatric Disorders	
Depression	Withdrawn patients; dry mouth; accelerated caries; oral candidiasis; chronic facial pain; burning mouth or sore tongue (oral dysaesthesia); occasionally temporomandibular pain dysfunction syndrome; halitosis; disturbed taste sensation
Chronic Fatigue Syndrome (CFS)	Unexplained fatigue; dry mouth is the only documented symptom in the oral cavity
Bipolar Disorder	Overactive, overtalkative, energetic in manic cycle; depressive disorder symptoms in the depressed cycle
Mania	Abnormal or excessive elation; excessively euphoric mood; unusual irritability; less need for sleep; grandiose notions and unrealistic beliefs in abilities and powers; excessive talking; racing thoughts, jumping from one idea to another (‘butterfly mind’); distractibility; excessive sexual desire; greatly increased energy and provocative, intrusive or aggressive behaviour; poor judgment; inappropriate social behaviour; spending sprees; abuse of drugs, particularly cocaine, alcohol and sleeping medications; denial that anything is wrong.
Schizophrenia	Loss of social contact; flatness of mood or inappropriate social behaviour which may appear at first as mere tactlessness or stupidity; auditory hallucinations – thought echoes or voices talking about the patient in the third person; passivity – the feeling of being controlled by external forces; thought insertion or withdrawal; thought broadcasting; delusions; hallucinations; tardive dyskinesia (TD)

## Results

Identification of mental health problems

The common psychiatric problems encountered in dental practice, as recognized and described by the American Dental Association (ADA) are anxiety disorders, mood disorders, psychotic disorders, and eating disorders [5]. Anxiety disorders include generalised anxiety disorder (GAD), obsessive-compulsive disorder (OCD), phobias, post-traumatic stress disorder (PTSD), mood disorders including bipolar disorder, major depressive disorder (MDD), psychotic disorders including schizophrenia, eating disorders like anorexia nervosa and bulimia nervosa. Mental health problems are classified as (i) identifiable brain disease (organic) and (ii) no obvious brain structural abnormality (functional), or (i) psychosis and (ii) neurosis. The ICD-10 covers all mental health and behavioural disorders from F00-F99 as listed in Table 1 [4]. Therapies for mental health problems include conventional psychotherapy, psychoactive medications, and cognitive behavioural therapy (CBT).

Identification of determinants that affect mental health

The determinants of mental health are neurotransmitters secreted in the brain like acetylcholine (ACh), epinephrine (adrenaline), dopamine, endorphins and enkephalins, gamma-aminobutyric acid (GABA), glutamate, neuropeptide Y, noradrenaline, oxytocin, phenylethylamine, serotonin (5-hydroxytryptamine, 5-HT), and substance P. There is also recent evidence that relates to neuroimmunomodulation and psychiatric disorders. Poor oral health also precipitates mental health disorders [6,7]. The imbalance in cytokine production namely interleukin (IL)-6, IL-1β, and interferon (INF)-γ with immunosuppression due to periodontal diseases also play a role in the etiopathogenesis of MDD. Understanding this is essential as periodontal diseases are to be effectively managed to prevent the further progression of existing mental health problems [8].

Identification of risk factors in patients with mental health problems in dentistry

The general and behavioural aspects of various mental health problems are listed in Table 1. This gives a clear picture of the requirement of dental and mental health providers to integrate tailored support to impart overall health and well-being by better patronization regardless of their immediate oral health requirements [9]. It is important to recognize the patient as a whole rather than having tunnel vision toward their dental needs only. Another challenge for these special needs patients is polypharmacy in psychiatry where two or more psychiatric medications are given to the patient [10]. This has widened the role of oral physicians to a broader spectrum of learning and keeping par with developments and guidelines in pharmacology while prescribing drugs in dental offices to avoid unwanted drug interactions for patients (Table 2). 

Identification of psychotropic drugs affecting oral health and its interactions

The commonly used drugs in psychiatric care [11-13] are antidepressants that include selective serotonin reuptake inhibitors (SSRIs), tricyclic antidepressants (TCA), monoamine oxidase inhibitors, antipsychotics that include typical (older type) antipsychotics and atypical (newer type) antipsychotics, anxiolytics or sedatives that includes benzodiazepines, beta-blockers, psychostimulants, and anticholinergic anti-parkinsonian drugs, and drugs for bipolar disorders that include carbamazepine, lamotrigine, lithium, and sodium valproate. The most common side effects of psychotropic drugs are xerostomia leading to candidiasis and dental caries by antidepressants and SSRIs, bruxism in antipsychotics and psychostimulants, post-surgical bleeding due to thrombocytopenia by sodium valproate, and hypersalivation with clozapine. Also, the interactions between psychotropic drugs and drugs commonly prescribed in dental practice are to be known.

Identification of psychotropic drugs that interact with common dental drugs

The common drugs prescribed in dental care are nonsteroidal anti-inflammatory drugs (NSAIDs), antibiotics, opioids, and tramadol. NSAIDs interact with sodium valproate and SSRI and prolong bleeding [14]. NSAIDs and antibiotics react with lithium, the common antipsychotic drug, to potentiate and increase its toxic defects by reducing renal clearance. Opioids and tramadol interact with antipsychotics or tricyclic antidepressants and lower the seizure threshold [15]. Monoamine oxidase inhibitors are not to be given with tramadol and pethidine as it increases the susceptibility of hypertension and other components of the autonomic nervous systems. Tramadol interacts with antidepressants and causes life-threatening serotonin syndrome due to the overactivation of 5HT-1A and 5HT-1B receptors with most patients reporting in emergency care within six hours [16]. The patients present with altered mental status, muscle rigidity, peripheral hypertonicity, and truncal rigidity leading to respiratory failure if untreated. The macrolides prescribed for dental infections interact with carbamazepine and valproate concentrations with synergistic action and increase drug levels in circulation by CYP3A4 inhibition [17]. Antibiotics increase lithium concentration by reducing renal clearance [18]. When carbamazepine is given concurrent with doxycycline, carbamazepine decreases the doxycycline half-life by up to 50% [19]. Macrolides interact with antipsychotics, tricyclic antidepressants, fluoxetine, or venlafaxine leading to QT prolongation [20].

## Discussion

There is a bidirectional relationship between dental and mental health [21]. Despite significant progress in dentistry with advanced technologies implemented in practice, patients with mental health problems do not get access to adequate oral health care. This is due to stigmas surrounding both the dentist and the patient when it comes to treating these special care patients. Also, various other barriers like dependency on family members for travel to the dentist's office and treatment expenses also exist. Dentists' ethical and moral professional duty is to evaluate them without preconceived notions and hesitations and welcome them for proper dental care in the same manner as any healthy individual. Dentists' lack of knowledge and skill in special needs dentistry has been considered a barrier to dental healthcare. Similarly, psychiatrists lack training in screening for oral health unless patients complain of dental symptoms verbally. Hence, it is the need of the hour to bridge this professional gap among practitioners of oral and mental health.

Preventive dentistry must be emphasized as most patients with mental health problems neglect oral healthcare, which draws subsequent dental problems. The dentist must undergo special care or special needs dentistry training in dealing with patients with mental health problems. Extreme empathy, patience, and tact are necessary with essential knowledge of adverse reactions to their common drugs, and potential legislative issues in treating them. The liaison between psychiatrists and dentists is necessary and consultation liaison models have to be created for treating patients with mental health problems. Hence, the proposed framework of Psychiatric Dental Consultation Liaison (PDCL) services is attaining success and popularity for a confident approach towards oral care delivery among dentists [22-23].

**Figure 1:**
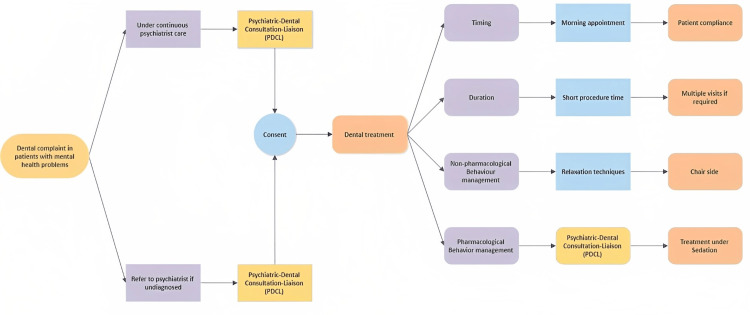
Algorithm in the management of dental patients with mental health problems in special care dentistry

In PDCL services, when a patient with a mental health problem reports a dental complaint, it is advised to include psychiatric consultation in effective behaviour management for better patient compliance and motivation toward treatment. The algorithm for using PDCL is described in Figure 1. This multisectoral approach would be more beneficial if the liaison exists among professional circles so that psychiatrists can also be made aware of the early detection of dental problems, and subsequent referrals to dental treatment when deemed necessary. Scheduled oral screenings are to be done every six months for patients with mental health problems irrespective of outpatient care or institutionalized care for the evaluation of oral hygiene status and to reduce the burden of disease progression. 

PDLC services apart from evaluation and issuing clearance by the psychiatrist for invasive procedures in dentistry also cover a range of services from behavioural relaxation techniques, antipsychotics, hypnotics, antidepressants, hypnosis, biofeedback, rational emotive behavioural therapy (REBT), CBT, and anxiolytics. This transparent consultation enables the patient or patient’s caregiver to choose between various behaviour modification techniques and thereby lessens the susceptibility to drug interactions. Additionally, this also overcomes the dentist’s barrier and stress in executing proper comprehensive care and boosts the quality of treatment performance.

Another valuable tool that needs to be included in the management of dental patients with psychiatric problems in special care dentistry is clinical audits [21]. This quality improvement process enables reporting the hurdles in treatment care with a candid and supportive approach so that new guidelines and protocols can be created for a conducive professional environment that is built upon compassion in delivering treatment care. The auditing can be prospective or retrospective. This includes topic preparation, selection criteria, measuring outcomes, improvement implementation, and sustaining growth [24].

The types of oral health interventions are educational, physical, a combination of behavioural and educational elements, and a combination of educational and physical elements [25]. However, no single intervention can suit all groups of individuals due to the diversity of mental health problems. To date, no policies have been issued by any governing bodies in India, which is the need of the hour. Therefore, an integrated, multidisciplinary, tailored toolkit is mandatory in approaching treatment planning for patients with mental health problems.

## Conclusions

We recommend PDCL services as an indispensable aid for psychiatric patients who require dental care. This framework supports a better understanding among oral physicians and clinical psychiatrists for early diagnosis of health issues and formulation of a proper treatment plan for the welfare of the patient without compromising their comfort. In special care dentistry, this professional liaison and mutual reciprocation eventually leads to complete successful treatment execution without breaks in appointment schedules for individuals with mental health disorders and thereby results in a heightened sense of physical, social, and psychological well-being. Also, future research has to be conducted examining the relationships between oral health-related quality of life [OHRQoL] and diagnosed mental health problems to explore further insights.
